# Recurring food source‐based *Listeria* outbreaks in the United States: An unsolved puzzle of concern?

**DOI:** 10.1002/hsr2.1863

**Published:** 2024-02-04

**Authors:** Ranjan K. Mohapatra, Snehasish Mishra, Lawrence Sena Tuglo, Ashish K. Sarangi, Venkataramana Kandi, Amani Ahmed AL Ibrahim, Hussain A. Alsaif, Ali A. Rabaan, Md. Kudrat‐E Zahan

**Affiliations:** ^1^ Department of Chemistry Government College of Engineering Keonjhar Odisha India; ^2^ School of Biotechnology KIIT Deemed University Bhubaneswar Odisha India; ^3^ Department of Nutrition and Dietetics, School of Allied Health Sciences University of Health and Allied Sciences Ho Ghana; ^4^ Department of Chemistry, School of Applied Sciences Centurion University of Technology and Management Balangir Odisha India; ^5^ Department of Microbiology Prathima Institute of Medical Sciences Karimnagar Telangana India; ^6^ Deparment of Pharmacy Jubail General Hospital Jubail Saudi Arabia; ^7^ Batterjee Medical College Jeddah Saudi Arabia; ^8^ Molecular Diagnostic Laboratory Johns Hopkins Aramco Healthcare Dhahran Saudi Arabia; ^9^ College of Medicine Alfaisal University Riyadh Saudi Arabia; ^10^ Department of Public Health and Nutrition The University of Haripur Haripur Pakistan; ^11^ Department of Chemistry Rajshahi University Rajshahi Bangladesh

**Keywords:** control measures, foodborne illness, food safety, HACCP, *Listeria* outbreak

## Abstract

Recurring *Listeria* outbreaks in the United States is a growing public healthcare concern. Although no associated reported death, 17 were hospitalized out of the 18 reported illnesses in the recent outbreak in 15 US states. The United States has experienced about 30 *Listeria* outbreaks in the last decade with 524 Listeriosis cases and 80 deaths. The identified origin were ice cream, leafy greens, mushroom, meat slice, dairy products like cheese, packaged salads, cooked chicken, hard‐boiled egg, pork product, frozen vegetable, raw milk, packaged caramel apple, bean sprout and soya products. Although rare, *Listeria* may lead to serious illness (invasive listeriosis) or death. Listeriosis is critically harmful and medically complicated, especially in the pregnant, the old above 65 years and in the immunocompromised. It could cause premature birth, miscarriage or even neonatal death. Hospitalization is often necessary in the geriatric, being fatal at times. Among *Listeria* sp., *Listeria monocytogenes* is often human infection‐associated. It is a gram‐positive, non‐sporulating, motile bacillus opportunistic pathogen. Food‐borne listeriosis is often associated with frozen foods due to its ability to thrive at low temperatures. Hypervirulent strains of *L. monocytogenes* with an ability to infect the respiratory system (the lungs) was recently reported in the coronavirus disease‐19 patients during the pandemic. *L. monocytogenes* seemed to have developed antimicrobial resistance to ciprofloxacin and meropenem, possibly acquired through the food chain. An early onset of listeriosis in the newborn is evident in the first 7 days postparturition. As the bacteria colonize the genitourinary tract, majority of such cases result from teratogenic transfer during vaginal delivery. Premature newborns, neonates born outside healthcare facilities and low‐birth‐weight babies were increasingly predisposed to an early onset of listeriosis. *Listeria* outbreaks were earlier reported in South Africa, Australia and Europe, with an unclear origin of the outbreaks. Social media updates about such outbreaks, the most likely food source, and measures to self‐protect are suggested as preventive measures. The article deals on various such aspects related to listeriosis primarily originating from food, to ensure better public healthcare and human wellness.

## 1 INTRODUCTION

The recent *Listeria* outbreak reports in the United States are concerning. It is worrying primarily for two reasons. One, aerobic bacterial food infections and intoxication cases are quite rare owing to prevailing typical anaerobic food packaging practices there, and two, the US food and pharmaceutical companies are globally known for their high standards in handling the microbiological hazards particularly (under the stewardship of the USFDA) in line with the ISO and HACCP compliance. Although no associated death is reported currently, 17 were hospitalized out of the 18 reported illnesses in 15 US states, the Centres for Disease Control and Prevention (CDC) reports.^1^ Studies on the specific food that is potentially at risk as the precursor of the outbreak and the yet‐to‐ascertain responsible factors are being undertaken. Identifying the potential food source behind the outbreak was jointly investigated by the CDC and the United States Food and Drug Administration (USFDA) which included interviewing the patients on the kind of food they consumed before falling sick. The CDC found that an ice cream manufacturing house and its warehouse in Brooklyn, NY were supposedly the source or origin of the recent outbreak.^2^ It was confirmed upon isolating the *Listeria* strain responsible for the outbreak from the “Soft Serve On The Go” vanilla chocolate cup ice cream that was invariably consumed by many patients. The NY State Department of Agriculture and Markets isolated *Listeria* strain from the ice cream cups at the manufacturing unit. An identical *Listeria* was also isolated from the environmental samples from vending stores of the ice cream.^2^ Leafy greens and various branded packaged salads were also consumed before falling sick, the CDC reported.^3^

A similar *Listeria* outbreak attributed to the vanilla ice cream was reported in Florida in 2022, of which 28 persons were hospitalized and one died.^4^ As per the CDC, the US experienced about 30 such *Listeria* outbreaks in the last decade, with a total reported 524 listeriosis cases and 80 deaths (15.26% case fatality rate, CFR).^5^ The transmission sources included ice cream, leafy greens, mushroom, meat slice, dairy products like cheese, packaged salads, cooked chicken, hard‐boiled egg, pork products, frozen vegetable, raw milk, packaged caramel apple, bean sprout, and soya products. The minimally processed food was potentially the source of *Listeria* and other food‐borne bacterial (like *Staphylococcus aureus* and *E. coli*) and viral (like Norovirus) pathogens, it was opined.^6^ The recurrence of *Listeria* outbreak in the United States surprises as the aerobic bacterial food infections and intoxications cases are expected to be rare owing to the prevailing typical anaerobic food packaging practices there. Also, the food and pharmaceuticals companies, under the strict monitoring of the USFDA have set high benchmarks in line with ISO and HACCP compliance, especially in containing microbiological hazards.

*Listeria* outbreak is not limited to the United States only as several such outbreaks with unclear origin in South Africa, Australia, and Europe are also documented. This article has made a detailed analysis of the compiled literature about the various reported *Listeria* outbreaks, its relation with the most likely food source and draws conclusions and recommendations to counter such possible outbreaks in future so that better public healthcare and human wellness is ensured.

## 2 MATERIALS

The materials for the article were formulated by considering relevant reports on *Listeria* outbreaks recurrence in the United States through a comprehensive search of online literature and acquiring the data published by the WHO, CDC, PubMed, ScienceDirect and Web of Science. The information from the selected articles was reviewed, shortlisted and compiled to develop an article with a seamless flow of events and observations for easy understanding of the readers and draw conclusions. The cited literature was compiled manually and presented in the references section of the article as per the Journal guidelines.

## 3 COMPLICATIONS

Although rare, *Listeria* may lead to serious illness (like invasive listeriosis) or death, as evidenced from the high (15.26%) CFR noticed during the United States outbreaks in the last decade. Listeriosis is reportedly critically hazardous and medically complicated in the pregnant, the geriatric above 65 years, and in individuals with weak immunity. Being fatal, hospitalization is often necessary in the geriatric. The affected cases for *Listeria* infection in the United States were primarily the age‐extremes (neonates and the geriatric) population, the pregnant and the immunocompromised.^7^, ^8^, ^9^ *Listeria* causes premature birth, miscarriage or even neonatal death. Listeriosis symptoms like fever, muscle ache, tiredness, headache, stiff neck, confused state of mind, loss of balance or seizures are manifested usually 2 weeks after infection. *Listeria monocytogenes* is often associated with human infection among the many *Listeria* sp.

A study evaluated the food‐borne infection risks in patients undergoing organ (kidney, liver, heart, and lungs) transplants in Switzerland between 2008 and 2018,^10^ and revealed that 88% of the confirmed microbial illnesses cases were due to *Campylobacter* and 10% were attributed to nontyphoidal *Salmonella*. Other isolated cases were attributed to *Shigella*, *Yersinia* and enteropathogenic *E*. *coli*. As all these pathogenic organisms are usually food‐borne, the study concluded that post‐transplant food‐borne ailment was a critical cause of morbidity and mortality in such patients. A potential *Listeria*‐induced endocarditis case was reported in a 74‐year‐old hemodialysis patient with bacteraemic *L. monocytogenes*, and^11^ F‐fluorodeoxyglucose‐positron emission tomography‐computed tomography confirmed endocarditis.^12^ Although *Listeria* is not a usual causative agent of infective endocarditis, the high‐risk immunosuppressed and the elderly are susceptible.^13^ *Listeria* epidemiology includes complex habitat like soil and water environment, and colonization in animals and humans. It causes invasive infections, especially in the debilitated and immunosuppressed (like transplant patients, pregnant women, individuals on long‐term immunosuppressive drugs and neonates), and can stay silently in a human body with no symptoms. *Listeria* could lead to sepsis, meningitis and neurolisteriosis initially, that may lead to severe morbidity and mortality without timely interventions.^14^, ^15^, ^16^, ^17^, ^18^

## 4 OUTBREAK CHARACTERIZATION

*Listeria* is a motile gram‐positive non‐sporulating bacillus. Believed to be an opportunistic pathogen, it is associated with infections like breast abscess, bacteraemia, meningoencephalitis and others.^11^, ^19^, ^20^ Due to its ability to thrive at low temperatures, frozen food is often associated with listeriosis. *Listeria* diagnosis is carried out through basic laboratory techniques, although it is complex and often lacks brevity.^21^ The motility, stress tolerance and adaptability of *L. monocytogenes* determine its virulence and aggressive invasion into the intestinal epithelial cells (that causes intestinal infection) and various other cell types.^22^, ^23^, ^24^ Hypervirulent *L. monocytogenes* strains with an ability to infect respiratory system (the lungs) was reported recently in a coronavirus disease‐19 (COVID‐19) pandemic patient.^25^ The whole genome sequencing (WGS) of *L. monocytogenes* isolated from cheese revealed that it was resistant to antimicrobials (AMR) like ciprofloxacin and meropenem, assumed to have acquired along the food chain.^26^

## 5 ASSOCIATED FOOD VEHICLES

The United States has witnessed *Listeria* outbreaks earlier too, causing grim, life‐threatening illnesses. Table 1 below is a summary of the numerous recently reported listeriosis outbreaks in the United States. Listeriosis was earlier linked primarily to the consumption of raw milk and its products.^27^ Food‐borne hazardous bacteria like *Listeria*, *Salmonella*, *E. coli* and *Campylobacter* are often found in raw unpasteurized milk.^28^ The CDC recommended to avoid eating, serving or selling the recalled organic and traditional frozen fruits and vegetables and their products for households, restaurateurs and retailers. The consumption of pasteurized dairy product like milk, soft cheese, ice cream and yogurt is suggested, wherein the hazardous microbes are supposedly killed. This is more critical for those at greater risk, like the pregnant, individuals below 5 years and above 65 years, and the immunocompromised. Epidemiological and laboratory evidence of the 2016 outbreak specified unskilled handling of frozen vegetables as the likely source behind that health‐emergency situation.^29^ The 2018 outbreak was linked to Deli ham.^30^ The 2020 outbreak was linked primarily to the hard‐boiled egg.^31^ Listeriosis was linked to Deli meat and pork products earlier too.^32^, ^33^

**Table 1:** A summary of the reported *Listeria* outbreaks in the United States and its likely origin in the past decade (2011–2023).

Year	Associated food product	Source/origin
2023	Ice cream, leafy greens	Manufacturer, vending store, environment
2022	Ice cream, mushroom, cheese	Restaurant food, stores/warehouse
2021	Packaged salads, cooked chicken	Frozen foods, manufacturer
2020	Deli meats, mushrooms	Manufacturer, packaged food
2019	Hard‐boiled egg	Manufacturer, packaged food
2018	Deli ham, pork products	Manufacturer, packaged food
2017	Raw milk, cheese	Manufacturer, packaged food
2016	Frozen vegetable, milk, salad	Frozen Foods, unpasteurized milk
2015	Cheese, ice cream	Manufacturer, packaged food
2014	Packed caramel apple, sprouted bean, dairy products	Manufacturer, packaged food
2013	Cheese	Manufacturer, packaged food
2012	Cheese	Manufacturer, imported & packaged food
2011	Cantaloupe/melon	Stores/warehouse

## 6 RISK FACTORS

The infection in the newborn and infants by a novel bacterium *Cronobacter sakazakii* was linked to their formula food consumption.^34^, ^35^ The organism was identified as an emerging opportunistic pathogen. An early onset of listeriosis in the newborn is evidenced by the presence of *Listeria* in the first 7 days postparturition. Majority of such cases result from teratogenic bacterial transfer to the newborn during vaginal delivery as the bacteria colonize the genitourinary tract.^36^ *Listeria* transmission from mother to child was documented wherein 40 pregnant women and their children were evaluated for listeriosis by culturing their blood and cerebrospinal fluid (CSF). Clinical (fever and abdominal pain) and laboratory findings (elevated C‐reactive protein in white blood cells and neutrophils) in the pregnant are potential listeriosis biomarkers.^37^ Clinical signs in the newborn include respiratory distress and fever along with laboratory findings of raised C‐reactive protein, and decreased glucose in the CSF. A French study on 189 neonates revealed that treating the listeriosis‐suspected pregnant with antibiotics could prevent teratogenic transmission.^38^ Pregnant lady exposed to a high‐risk diet and exhibiting influenza‐like illness may be identified and administered with antibiotics like amoxicillin.^39^, ^40^ Premature newborns, neonates born outside the healthcare facilities and low‐birth‐weight babies were increasingly at risk for an early onset of listeriosis.^41^

Bovine spongiform encephalopathy (BSE), also known as the mad cow disease is a central nervous system (CNS) disease caused by prion protein. It is a pathologically transformed normal human protein affecting the brain. Infectious prion proteins survive high temperature and warrants effective screening during food processing.^42^ Many other foodborne diseases in food processing/manufacturing establishments pose a biohazard risk. Threatening foodborne pathogens include *E. coli*, *S. aureus*, *Campylobacter*, *Vibrio cholerae*, *V. parahemolyticus* and *Bacillus cereus* among the numerous others. As *Listeria* resists low temperature, it could survive especially in the reasonably highly perishable processed, packaged and stored foods at low temperature through the supply chain for enhanced shelf‐life. This enables the spread of *Listeria*, resulting in frequent food‐borne outbreaks. Studies have identified a *Listeria* serovariants shift from serovar 4b in the 1990s to serovar 1/2a in the 2000s.^43^ While assessing bulk‐tank bovine milk, a Greek study found that 1/2a and 3a serovariants were prevalent. Several virulence genes like *inl*A, *inl*C, *inl*J, *iap*, *plc*A, *hly*A and *act*A occurred in these isolates.^44^ That all isolated *Listeria* strains were multi‐drug resistant is alarming. Deciphering *Listeria* ecosystem and evaluating its transmission dynamics is important. It is suggestible to use whole genome sequencing (WGS), metagenomics and bioinformatic approaches to assess epidemiological characteristics, antimicrobial resistance and virulence gene markers.^45^

## 7 SUGGESTED RECOMMENDATIONS

Although *Listeria* outbreaks in South Africa, Australia and Europe were previously reported, the exact food source that was behind it has been unclear.^46^, ^47^, ^48^ Recommendations to counter *Listeria* infections in the pregnant, the newborn, the geriatric above 65 years, the immunosuppressed individuals, and the patients with cancer, AIDS, organ transplant and nephrological clinical conditions were proposed.^49^ They include hand‐washing after handling fruits and vegetables and ready‐to‐eat food, washing the unprocessed raw fruits and vegetables under running water, maintaining processed food either below 5°C or above 60°C, storing the packed and cooked meat separately and timely discarding the unconsumed refrigerated and packaged foods.^49^ *Listeria* could survive for up to 7 months (in bulk milk) to 7 years (in ice‐cream), as per a recent report on various food sources. Clearly, demarcating the hygienic‐handling (high‐risk) and other environs (low‐risk) zones in food processing facilities is suggested. Meticulous environmental screening to minimize the transfer of *Listeria* into the high‐risk zone resulting in the contamination of packaged food is suggested.

Molecular techniques like real‐time nucleic acid sequence‐based amplification (NASBA), PCR, loop‐mediated isothermal amplification (LAMP) and oligonucleotide‐based microarrays, as also immunological methods like immunomagnetic capture and lateral flow immunoassay/immunochromatography are useful state‐of‐the‐art screening and diagnostic tools. Optical, piezoelectric, electrochemical and cell‐based biosensor approaches and spectrophotometric methods like near‐infrared spectroscopy (NIR), Raman spectroscopy, matrix‐assisted laser desorption ionization time‐of‐flight mass spectrometry (MALDI‐TOF MS) and hyperspectral imaging in manufacturing unit and surrounding environments are also recommended for effective screening.^50^, ^51^, ^52^ Applying the thermal, non‐thermal, chemical, phyto‐extracted listeriostatic and listericidal agents to control *Listeria* is suggested. Using biopreservatives like essential oils, biological agents like *Lactobacillus* species, *Enterococcus faecium*, *Staphylococcus xylosus*, *Debaryomyces hansenii* and *Penicillium chrysogenum*, probiotics, and spices to inhibit the growth of pathogenic and to prevent microbial spoilage while retaining benign microbes in food packaging during manufacturing in line with the Hazard Analysis and Critical Control Point (HACCP) approach are suggested.^53^, ^54^

## 8 SAFETY ISSUES

Retailers need to clean and sanitize food‐display drawers, refrigerator shelves and freezers to avoid contamination. The USFDA recommends extra vigilance in cleaning and sanitizing food‐contact surfaces that may be in direct or indirect contact with contaminated food products to reduce the risk of cross‐contamination. Retailers and restaurateurs also need to sanitize and disinfect the food preparation, storage and serving areas to reduce the risk. At the same time, it is necessary that manufacturers strictly follow their in‐house hygiene and safety measures as per their laid down SSOP (Sanitation Standard Operating Procedure) and further strengthen it for effective biohazard control. Rapid and timely updates on social media about such outbreaks, the most likely food source to remain vigilant about or even avoid, and self‐protection measures from time to time are also suggested. Similar platforms could also be used to publish listeriosis‐specific clinical features, symptoms and likely transmission chain for larger public reach.

## 9 CONCLUSION

The United States has witnessed *Listeria* outbreak with life‐threatening consequence as discussed above. Due to typical anaerobic food packaging practices that prevail in the United States, recurring *Listeria* outbreaks in there certainly surprises. Under the USFDA monitoring, companies there maintain high standards, particularly in the food and pharmaceutical sectors, especially in containing microbiological hazards. Teratogenic listeriosis during vaginal delivery is a matter of concern. It is suggested to assess the epidemiological aspects, identify the gene markers of virulence and assay the antimicrobial resistance using whole genome sequencing (WGS), metagenomics and bioinformatics approaches, as well as numerous state‐of‐the‐art techniques as suggested above. Hygiene and sanitation seem to play a critical role in nipping the ailment at bud. Further, religiously following the USFDA‐set guidelines and maintaining high HACCP standards shall ensure that the process is fool‐proof throughout the food supply chain. All in all, the Quality Management System (QMS) needs to abide by in true letter and spirit to effectively to specifically counter listeriosis and numerous other such foodborne microbiological hazards in general.

## AUTHOR CONTRIBUTIONS

**Ranjan K. Mohapatra**: Conceptualization; project administration; writing—original draft. **Snehasish Mishra**: Writing—review & editing. **Lawrence Sena Tuglo**: Writing—original draft. **Ashish K. Sarangi**: Writing—original draft. **Venkataramana Kandi**: Writing—original draft. **Amani Ahmed AL Ibrahim**: Writing—review & editing. **Hussain Abdulkhaliq Alsaif**: Writing—original draft. **Ali A. Rabaan**: Supervision; Writing—review & editing. **Md Kudrat‐E Zahan**: Writing—review & editing.

## CONFLICT OF INTEREST STATEMENT

The authors declare no conflict of interest.

## TRANSPARENCY STATEMENT

The lead author Venkataramana Kandi, Md. Kudrat‐E Zahan affirms that this manuscript is an honest, accurate, and transparent account of the study being reported; that no important aspects of the study have been omitted; and that any discrepancies from the study as planned (and, if relevant, registered) have been explained.

## Data Availability

Not applicable.
